# Epigenetics-Associated Risk Reduction of Hematologic Neoplasms in a Nationwide Cohort Study: The Chemopreventive and Therapeutic Efficacy of Hydralazine

**DOI:** 10.3389/fonc.2022.809014

**Published:** 2022-02-02

**Authors:** Bing-Heng Yang, Wei-Zhi Lin, Yu-Ting Chiang, Yeu-Chin Chen, Chi-Hsiang Chung, Wu-Chien Chien, Chia-Yang Shiau

**Affiliations:** ^1^ Graduate Institute of Medical Sciences, National Defense Medical Center, Taipei, Taiwan; ^2^ Division of Clinical Pathology, Department of Pathology, Tri-Service General Hospital, Taipei, Taiwan; ^3^ Graduate Institute of Life Sciences, National Defense Medical Center, Taipei, Taiwan; ^4^ Fidelity Regulation Therapeutics Inc., Taoyuan City, Taiwan; ^5^ Department of Biochemical Science and Technology, National Taiwan University, Taipei, Taiwan; ^6^ Division of Hematology and Oncology, Department of Medicine, Tri-Service General Hospital, National Defense Medical Center, Taipei, Taiwan; ^7^ School of Public Health, National Defense Medical Center, Taipei, Taiwan; ^8^ Department of Medical Research, Tri-Service General Hospital, Taipei, Taiwan; ^9^ Taiwanese Injury Prevention and Safety Promotion Association, Taipei, Taiwan

**Keywords:** hematologic neoplasms, hydralazine, epigenetic drug, cohort, drug repositioning

## Abstract

**Background:**

Although several epigenetic drugs have been reported to have therapeutic efficacy for some hematologic neoplasms (HNs) in clinical trials, few achieved disease-free survival benefit. The traditional drug discovery pathway is costly and time-consuming, and thus, more effective strategies are required. We attempted to facilitate epigenetic drug repositioning for therapy of HNs by screening the Human Epigenetic Drug Database (HEDD) in the web, conducting a bench-work cytotoxicity test and a retrospective nationwide cohort study prior to a clinical trial.

**Methods:**

Four FDA-approved epigenetic drugs with antitumor properties and completion of clinical phase II trials were selected from HEDD. Hydralazine (HDZ) and valproate (VAL) among the four were selected with higher cytotoxicity to HN cells, no matter whether carrying the *JAK2V617F* mutation or not. Both of them were chosen for a cohort study using the Longitudinal Health Insurance Database (LHID) 2000–2015 (N = 1,936,512), a subset of the National Health Insurance Research Database (NHIRD, N= 25.68 millions) in Taiwan.

**Results:**

In the initial cohort, HDZ or VAL exposure subjects (11,049) and matching reference subjects (44,196) were enrolled according to maximal daily consumption (300/2,100 mg per day of HDZ/VAL). The HN incidence in HDZ and VAL exposure groups reduced from 4.97% to 3.90% (*p* <.001) and 4.45% (*p* = .075), respectively. A further cohort study on HDZ at a lower range of the WHO defined daily dose (<34 mg per day) and HN incidence of HDZ exposure subjects (75,612) reduced from 5.01% to 4.16% (*p* = 1.725 × 10 ^-18^) compared to the reference subjects (302,448).

**Conclusions:**

An association of a chronically prescribed HDZ, even prescribed low dose, with reduction of overall incidence rate and in most subgroups of HN was observed in our study. Repositioning HDZ for HN management may be feasible. This is the first nationwide cohort study of the epigenetics-associated risk evaluation of overall HN in the existing literature, showing an effective method with a wider scope to inform contemporary clinical trials of epigenetic drugs in the future.

## Introduction

Hematologic neoplasms (HNs) including Hodgkin lymphoma, non-Hodgkin lymphoma (NHL), leukemia, and multiple myeloma (1) show high prevalence in many countries. In 2016, there were 1,139,000 newly diagnosed HN cases and 677,000 deaths worldwide. From 2006 to 2016, the incidence of leukemia increased by 26% and that of NHL by 45% (2). Traditionally, chemotherapy is used as the first-line treatment for HN, but its therapeutic efficacy is limited and has various distressing side effects. In addition, long-term treatment with HN can cause significant financial burden on patients and their families, leading to an increased risk of mental illness and impact on life quality that may negatively affect the treatment outcome (3–5).

In cancer cells, genetic instability and epigenetic instability are observed (6). Cell activities such as cell proliferation, apoptosis, invasion, and senescence can be modulated by these modifications, and epigenetic dysregulations led to tumorigenesis (7). Drugs targeting epigenetic regulators have been tried clinically on some HN since 2004 (8). Epigenetic treatment may restore chemosensitivity in relapsed/refractory patients by reversing drug resistance to chemotherapy (9–12), or trigger an innate immune response by alter the function of relevant immune cells to acquired immunity (13).

Drugs, which have epigenetic targets, may be prescribed for indications other than HN. The Taiwan FDA has approved two epigenetic drugs: hydralazine (HDZ), an antihypertension drug shown to act as a DNA methyltransferase inhibitor (DNMTi), and valproate (VAL), an anticonvulsant and established histone deacetylase inhibitor (HDACi). Although clinical trials were conducted on both epigenetic drugs, no cohort study has been conducted prospectively or retrospectively to evaluate the association between HN incidence and the use of two drugs. Here, we carried out a cohort study respectively by analyzing the registries from the National Health Insurance Research Database (NHIRD) in Taiwan during 2000–2015 (14). A highly significant association between a low dose of HDZ and reduction of incidence was found for several subgroups of HNs. We observed a significant overall reduction in the incidence of most HNs among patients given with HDZ and in dosage-stratified analysis. Our results suggest it may be feasible to reposition HDZ for treating HN. Our cohort study shows a method with a wider scope to inform contemporary clinical trials of epigenetic drugs in the future. We propose that in view of the highly significant association with sufficient subjects, whether HDZ prescription to HN patients needed to be validated by clinical trial may be reconsidered.

## Methods

### Study Design and Drug Selection

**Figure 1:**
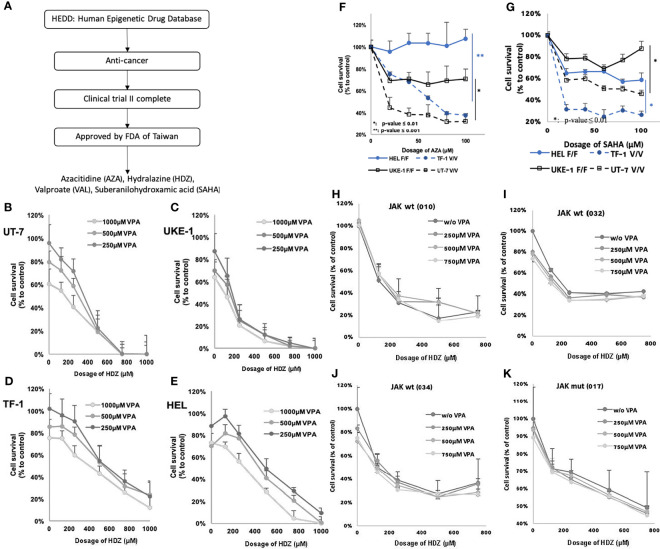
Flowchart showing steps for screening candidate drugs, from *in silico* and *in vitro* to *ex vivo*. **(A)** *In-silico* screening led to four candidate drugs AZA, HDZ, VAL, and SAHA. **(B–E)** JAK2V617F mutation may affect the therapeutic efficacy of AZA and SAHA, but not of HDZ-VAL. The cell survival of four MPN cell lines treated with HDZ-VAL combination. **(F, G)** The cell survival rate of four MPN cell lines treated with AZA or SAHA. **(H–K)** The inhibitory efficacy of cell survival rate after HDZ-VAL combinational treatment on the leukocytes of PV or ET patients.

Four epigenetic drugs were selected from the 64 listed in the Human Epigenetic Drug Database (HEDD) (15) because they have been reported to have antitumor activity and have passed clinical phase II trials to prove their safety. The four drugs chosen were azacitidine (AZA) and HDZ, both DNMTi, and VAL and suberanilohydroxamic acid (SAHA), both HDACi. These four candidate epigenetic drugs were preliminarily tested for their cytotoxicity. HDZ and VAL showed higher cellular toxicity, no matter whether carrying the *JAK2V617F* mutation or not (**Figure 1**). They were, therefore, selected for retrospective cohort studies for association with incidence reduction of all subgroups of HNs. To study the dose effect, the dose range was stratified according to the defined daily dose (DDD).

### Ethics

The personal identification data from NHIRD were encrypted to protect privacy. The protocol of this study was reviewed and approved by the IRB of the Tri-Service General Hospital (No.: 2-108-05-107, B-109-38)

### Isolation of Leukocytes From Clinical Waste Blood

Leukocytes were isolated from clinical waste blood of patients who received therapeutic phlebotomy. Briefly, the whole blood was 1-fold diluted with PBS. Tubes (50 ml) containing Ficoll-Paque PLUS solution (10 ml) were prepared beforehand. Diluted blood (25 ml) was slowly added into the tubes without stirring the under-layer solution. Then, the layered solution was centrifuged at 600 × g at room temperature for 40 min. The buffy coat in the middle layer was carefully transferred to a new tube. The cells of the buffy coat were washed with PBS and cultured in RPMI 1640 medium supplied with 10% fetal bovine serum, 2 g/l sodium bicarbonate, 2.5 g/l D-glucose, and 110 mg/l sodium pyruvate.

### Cytotoxicity Study

The UT-7 (ACC 137, RRID: CVCL_2233) cell line was purchased from DSMZ (Braunschweig, Germany). The HEL (HEL 92.1.7, RRID: CVCL_2481) cell line was obtained from American Type Culture Collection (ATCC, VA, USA). The UKE-1 (GM23245 B, RRID: CVCL_0104) cell line was purchased from Coriell Institute (Camden, NJ, USA). The TF-1 (RRID: CVCL_0559) cell line was kindly given by Dr. Jeffrey Jong-Young Yen (Institute of Biomedical Sciences, Academia Sinica, Taipei, Taiwan) and identified as a 100% match with the STR profile recorded in DSMZ (German Collection of Microorganisms and Cell Cultures GmbH, Braunschweig, Germany) (The result of STR profile is not shown).

UT-7, UKE-1, and HEL cells were cultured in RPMI 1640 medium supplied with 10% fetal bovine serum, 2 g/l sodium bicarbonate, 2.5 g/l D-glucose, 110 mg/l sodium pyruvate, and 0.5 µg/ml Mycoplasma Removal Agent (MP Biomedicals, Illkirch, France). TF-1 cells were cultured in RPMI 1640 medium under similar conditions and additionally supplied with 2 ng/ml GM-CSF. Cells were seeded onto 96-well plates at a density of 10,000 per well with/without exposure to candidate drugs. After incubation for 3 days, 10 µl of MTT reagent (1 mg/ml) was added into each well and incubated for 4 h. Then, 37% formaldehyde solution was added (final concentration 4%), and cells were sedimented by using centrifugation at 1,000 × g for 10 min at room temperature. The medium was removed carefully, and the formazan crystal was dissolved in 50 µl of DMSO. The absorption at 570 nm was read by an ELISA reader. The read was marked as *T_i_* for the test and *C* for that with no drug. The cell viability was calculated by the following formula:


$$Cell\;viability=T_{i}/C\;\times \;100\text{\% }Cell\;viability=T_{i}/C\;\times \;100\%$$

### Data Source

The National Health Insurance (NHI) Program was started in Taiwan in 1995 and enrolled more than 99.9% of the Taiwan population, by the end of 2014, according to Liang-Yu Lin (16). NHIRD is a nationally representative cohort that contains detailed registry and claims data from all 23 million residents of Taiwan, including outpatient departments and the inpatient hospital care settings. NHIRD collects basic demographic information, including gender, birthday, insurance premium, prescriptions, operations, investigations, medical encounters, and disease diagnoses according to International Classification of Diseases, 9^th^ Revision, Clinical Modification (ICD-9-CM) codes. All diagnoses of HNs in Taiwan are made by board-certified clinicians. The Bureau of NHI (a single-payer health insurance system) respectively and randomly reviewed the medical records of 1 in 100 ambulatory care visits and 1 in 20 inpatient claims to verify the accuracy of the diagnoses and appropriate management (see dataset link in section of *Data Availability Statement*). While only a small number of validation studies with small sample sizes have been undertaken, they have generally reported positive predictive values of over 70% for various diagnoses (17, 18). To date, over 2,700 peer-reviewed studies have been published using NHIRD data. Longitudinal Health Insurance Database (LHID) 2000–2015, a subset of the NHIRD (14), was used for this study. LHID 2000–2015 contains all the original claim data of 1,936,512 registries enrolled in year 2005 randomly sampled from the year 2005 Registry for Beneficiaries (ID) of the NHIRD. There are approximately 25.68 million individuals in this registry. There was no significant difference in the age distribution, average insured payroll-related amount, or gender distribution (χ^2^ = 0.008, df = 1, *p* value = 0.931) between the patients in the LHID 2000–2015 and the original NHIRD.

### Sample Selection

Patients who were treated with either HDZ or VAL continuously for more than 180 days during 2000–2015 were selected and assigned to the exposure group. Enrolling subjects in the exposure group was based on the drug prescriptions presented in medical records or patients’ profile. The index date refers to the date that the patient was registered in the database; follow-up began after this date. The inclusion date is defined as day 181 after successive exposure to HDZ or VAL. Subjects, who had been treated with HDZ or VAL before the index date, had been treated with HDZ and VAL simultaneously, had HN before tracking, had lost proper records of their HDZ or VAL dosage and duration, and were younger than 20 years or whose gender was not known were all not included. Exposure groups that were treated with HDZ or VAL were 1:1 matched and referred to as the “HDZ group” and “VAL group”, respectively. Subjects without HDZ and VAL exposure were 4:1 matched to HDZ and VAL exposure groups and served as a reference group. All the subjects were matched according to baseline variables such as age, gender, and index date.

To investigate the effect of the dosage of HDZ or VAL on the association with HN incidence, a stratified analysis was applied initially using three levels of dosage: 0%–33%, 34%–66%, 67%–100% of DDD, which is 2,000 mg per day for VAL and 300 mg per day for HDZ, according to maximal daily consumption. In a further HDZ cohort study, a lower level of dose range, 100 mg per day, according to WHO DDD guidance, was analyzed.

### Covariates

The covariates included gender, age groups (20–29, 30–39, 40–49, 50–59, and over 60 years old), educational levels (under 12 or over 12 years), marital status (married or unmarried), healthcare insurance premium [<18,000, 18,000–34,999, ≥35,000 New Taiwan Dollars (NT$)], seasons, location of residence (north, center, south, and east of Taiwan), urbanization level of residence (levels 1 to 4), and level of hospital (medical center, regional hospital, or local hospital). The urbanization level of residence was defined based on population and several indicators of developmental level. Level 1 was defined in areas containing a population more than 1,250,000 with a specific designation of political, economic, cultural, and metropolitan development. Level 2 was defined as a population in the range of 500,000 to 1,249,999 with an important role in politics, economy, and culture. Level 3 and level 4 were defined as a population in the range 150,000 to 499,999 and under 149,999, respectively.

The comorbidities included hypertension (HTN, ICD-9-CM 401-405), gestational HTN (ICD-9-CM 642.0-642.3, 642.7, 642.9), idiopathic pulmonary artery hypertension (IPAH, ICD-9-CM 416.0), congestive heart failure (CHF, ICD-9-CM 428), affective psychosis (ICD-9-CM 296), epilepsy (ICD-9-CM 345), migraine (ICD-9-CM 346), pulmonary embolism (PE, ICD-9-CM 415.1), gastric ulcer (ICD-9-CM 531), peptic ulcer disease (PUD, ICD-9-CM 533), gastrojejunal ulcer (ICD-9-CM 534), gastrointestinal hemorrhage (ICD-9-CM 578), Budd-Chiari syndrome (ICD-9-CM 453.0), cerebral thrombosis (ICD-9-CM 434.0), ischemic heart disease (IHD, ICD-9-CM 411), vascular insufficiency of intestine (ICD-9-CM 557), obesity (ICD-9-CM 278), and hepatitis B virus (HBV, ICD-9-CM 070.2-070.3). The Charlson Comorbidity Index after removal of the aforementioned comorbidities and malignancies (CCI_R) was used to evaluate the extent of the comorbidities.

### Outcome Measure

The HNs described in this study follow the ICD guidelines for hematological disorders: (1) lymphosarcoma and reticulosarcoma and other specified malignant tumors of lymphatic tissue (ICD-9-CM 200); (2) Hodgkin’s disease (ICD-9-CM 201); (3) other malignant neoplasms of lymphoid and histiocytic tissue (ICD-9-CM 202); (4) multiple myeloma and immunoproliferative neoplasms (ICD-9-CM 203); (5) lymphoid leukemia (ICD-9-CM 204); (6) myeloid leukemia (ICD-9-CM 205); (7) monocytic leukemia (ICD-9-CM 206); (8) other specified leukemia (ICD-9-CM 207); (9) leukemia of unspecified cell type (ICD-9-CM 208); (10) MPN-like neoplasm (ICD-9-CM 238.4-polycythemia vera; ICD-9-CM 238.5-neoplasm of uncertain behavior of histiocytic and mast cells; ICD-9-CM 238.6-neoplasm of uncertain behavior of plasma cells; ICD-9-CM 238.71-essential thrombocythemia); (11) MDS (ICD-9-CM 238.72-low grade myelodysplastic syndrome lesions; ICD-9-CM 238.73-high grade myelodysplastic syndrome lesions; ICD-9-CM 238.74-myelodysplastic syndrome with 5q deletion; ICD-9-CM 238.75-myelodysplastic syndrome, unspecified; ICD-9-CM 238.76-myelofibrosis with myeloid metaplasia; ICD-9-CM 238.79-other lymphatic and hematopoietic tissues; ICD-9-CM 289.83-myelofibrosis); (12) paraproteinemia (ICD-9-CM 273.1-monoclonal paraproteinemia; ICD-9-CM 273.2-other paraproteinemias; ICD-9-CM 273.3-macroglobulinemia; ICD-9-CM 273.8-other disorders of plasma protein metabolism; ICD-9-CM 273.9-unspecified disorder of plasma protein metabolism); and (13) other polycythemia (ICD-9-CM 289.0-polycythemia, secondary; ICD-9-CM 289.6-familial polycythemia) (**Table S1**).

The 1^st^ endpoint in the cohort was defined as the time to stop follow-up when the diagnosis of any HN was made for a subject; while the 2^nd^ endpoint was defined as the end of tracking when follow-up of all the subjects was stopped. To prevent multiple counting of HN on each subject, the HR of overall HR was not calculated at the 2^nd^ endpoint.

### Statistical Analysis

SPSS software v.22.0 (IBM Corp., Armonk NY, USA) was used for all analysis. The chi-square test was used to compare categorical variables by different treatment type when the categorical outcomes were larger than 5 and Fisher’s exact test was used when the categorical outcome was smaller than 5. One-way ANOVA with Scheffe *post-hoc* test was used to compare continuous variables. The Kaplan–Meier method and log-rank test with follow-up time as time scale were used to evaluate the difference of cumulative incidence of HNs between the three groups. Multivariate Cox regression analysis adjusted by covariates mentioned above was used to determine the association of HNs, and the results were presented as a hazard ratio (HR) with a 95% confidence interval (CI). A two-tailed *p* value of less than 0.001 was considered statistically significant (19). We used Schoenfeld’s global test to evaluate proportional-hazards assumption (20).

In order to verify the substantial therapeutic potential of the drugs, a sensitivity analysis which excluded the patients earlier diagnosed with HNs after the tracking year was performed. For causal analysis of competing risks, the Fine and Gray competing risk model with all-cause mortality as variant was used to confirm competing risk of mortality (21).

## Results

### Candidate Drug Selection From the Human Epigenetic Drug Database

A total of 64 epigenetic drugs were listed in the HEDD. Thirty-three of them have been studied for cancer therapy. Listed drugs which were still under development in preclinical or clinical phase I or II trials were excluded (**Table S2**). Finally, four epigenetic drug candidates were selected from HEDD because they had been reported to have antitumor activity and had passed a phase II clinical trial to prove their safety. The drugs chosen were two DNMTi—AZA and HDZ, and two HDACi—VAL and SAHA (**Figure 1A**). Furthermore, HDZ and VAL among the above four drugs were found to exert stronger cytotoxicity on HN cell lines and on leukocytes prepared from clinical discarded blood over therapeutic phlebotomy, in comparison with SAHA and AZA which showed much less cytotoxicity (**Figures 1B–K**). Thus, HDZ and VAL were chosen for the present retrospective cohort study.

### Sample Selection Criteria and Flowchart

**Figure 2:**
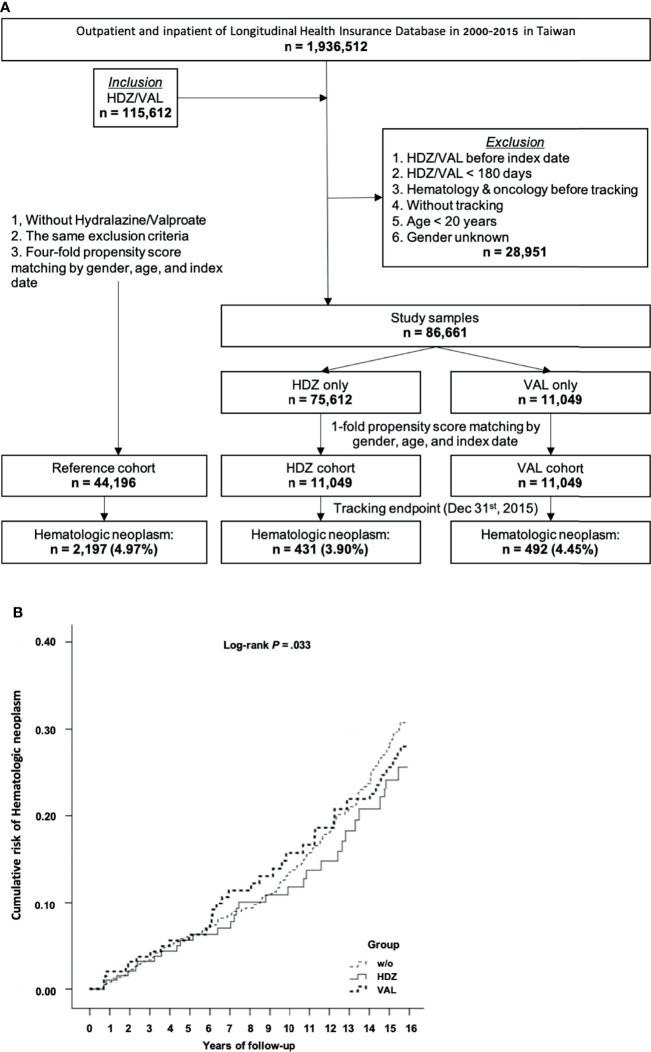
A cohort study for association of HN incidence reduction with HDZ or VAL. **(A)** A flow chart showing the steps for sample-subject selection for the cohort study based on chronic prescription of HDZ and VAL. **(B)** The Kaplan–Meier curve of the cumulative incidence of being diagnosed with HN among the different groups with log-rank test. Log-rank test: without vs. hydralazine, *p* <.001; without vs. valproate, *p* = .067; hydralazine vs. valproate, *p* = .075.

Among a total of 1,936,512 outpatient and inpatient registries of the NHIRD in Taiwan during 2000–2015, 115,612 subjects were enrolled. Of these, 28,951 subjects were excluded based on the criteria listed in *Material and Methods*. Of the remaining 86,661 subjects, 75,612 subjects were matched to the HDZ exposure group and 11,049 were matched to the VAL exposure group. Initially, the number of subjects matching the VAL exposure group (11,049) was adopted for both HDZ and VAL exposure group for the purpose of comparing the potential association between their prescription and HN incidence. The 4-fold (44,196) subjects were randomly selected as the reference group from the non-exposure subjects, respectively (**Figure 2A**). The stratified higher DDD of HDZ (300 mg per day) and VAL (2,100 mg per day), referring to maximal daily consumption, was initially adopted for comparison. Later, a further cohort study focusing on a lower level of dose range of HDZ (100 mg per day) following the WHO DDD guidance was analyzed since HDZ was associated with a much higher reduction of HN incidence than VAL. The dose effect was illustrated by stratification of dose into three levels: high (≧67%), intermediate (34%–66%), and low (<34%).

### Characteristics of the Study Population

Covariates such as gender, age, marital status, educational level, healthcare insurance premium, comorbidities, season, location of residence, urbanization level of residence, and level of hospital were taken as baseline. Overall, percentage of male subjects was a little higher but not significant than that of female. The proportion of subjects aged 60 years and older is approximately 57%. While about one-third of subjects lived in the north part of Taiwan or high urbanization level of region, the index of hospital level was about equally distributed from medical center to local hospital. Comorbidities such as HTN, affective psychosis, gastrojejunal ulcer, Budd-Chiari syndrome, IHD, and HBV are independent risk factors. Nevertheless, the CCI_R ratio was positively associated with the incidence of HN (**Tables S3**, **S4**).

### The Association of HN Incidence

In this initial cohort study, the association of HN incidence with HDZ and VAL exposure was compared in contrast to the reference group. At the endpoint of follow-up, 431 subjects (3.90%) in the HDZ exposure group while 492 subjects (4.45%) in the VAL exposure group were diagnosed with HN. However, up to 2,197 subjects (4.97%) were diagnosed with HN in the reference group (**Figure 2A**). After adjusting for the covariates by the multivariate Cox regression analysis, the adjusted HRs of being diagnosed with HN were recorded and are presented in **Table 1**. The adjusted HR of the HDZ exposure group to overall HN was significantly lower than that of the reference group (*p* <.001, 95% CI: 0.522–0.840), but the VAL exposure group showed no significant difference (*p* = .075, 95% CI: 0.642–1.032).

**Table 1:** Adjusted HR of HN according to subgroup stratified by dose level of HDZ and VAL.

Hematologic Neoplasm Subgroup	Drug, Dose (DDD *^b^*)	1^st^ Endpoint^a^				2^nd^ Endpoint^a^			
		Adjusted	95% CI		*p*	Adjusted HR	95% CI		*p*
		HR							
Overall	Without	Reference							
	Hydralazine	0.714	0.522	0.840	2.57E-12				
	<34%	0.772	0.565	0.908	2.98E-11				
	34%–66%	0.713	0.522	0.839	3.36E-12				
	≥67%	0.569	0.416	0.669	4.06E-13				
	Valproate	0.877	0.642	1.032	.075				
	<34%	0.909	0.665	1.070	.106				
	34%–66%	0.885	0.648	1.042	.089				
	≥67%	0.781	0.571	0.989	.041				
Lymphosarcoma and reticulosarcoma	Without	Reference				Reference			
	Hydralazine	0.728	0.533	0.857	3.57E-6	0.737	0.540	0.868	3.64E-6
	<34%	1.515	0.908	1.782	.202	1.534	0.919	1.804	.225
	34%–66%	0	–	–	.998	0.812	0.597	0.972	5.78E-7
	≥67%	0	–	–	.999	0.675	0.317	0.875	4.03E-8
	Valproate	1.175	0.860	1.382	.187	1.190	0.871	1.399	.208
	<34%	1.631	0.943	1.919	.371	1.651	0.955	1.943	.413
	34%–66%	1.186	0.868	1.395	.190	1.201	0.879	1.412	.211
	≥67%	0	–	–	.999	0.986	0.718	1.285	.371
Hodgkin’s disease	Without	Reference				Reference			
	Hydralazine	0.642	0.470	0.756	2.79E-6	0.650	0.476	0.765	4.82E-6
	<34%	0.891	0.652	1.048	.065	0.902	0.660	1.061	.072
	34%–66%	0.649	0.475	0.763	4.50E-7	0.657	0.481	0.772	5.96E-6
	≥67%	0	–	–	.997	0.524	0.311	0.670	8.06E-5
	Valproate	1.152	0.843	1.355	.246	1.166	0.853	1.372	.274
	<34%	1.439	0.953	1.693	.201	1.457	0.965	1.714	.224
	34%–66%	1.395	0.921	1.641	.190	1.412	0.932	1.661	.211
	≥67%	0	–	–	.998	1.086	0.771	1.246	.295
Other malignant neoplasms of lymphoid and histiocytic tissue	Without	Reference				Reference			
	Hydralazine	0.634	0.464	0.746	8.83E-10	0.626	0.458	0.737	2.67E-9
	<34%	0.681	0.498	0.801	6.25E-9	0.673	0.492	0.791	9.33E-9
	34%–66%	0.620	0.453	0.729	5.37E-10	0.612	0.447	0.72	7.82E-10
	≥67%	0.540	0.395	0.635	4.24E-11	0.533	0.390	0.627	6.61E-11
	Valproate	0.858	0.628	1.010	.054	0.848	0.620	0.998	.049
	<34%	0.916	0.670	1.078	.073	0.905	0.662	1.065	.066
	34%–66%	0.866	0.634	1.019	.059	0.855	0.626	1.007	.053
	≥67%	0.697	0.501	0.940	.042	0.689	0.504	0.929	.038
Multiple myeloma and immunoproliferative neoplasms	Without	Reference				Reference			
	Hydralazine	0.578	0.423	0.680	7.86E-8	0.571	0.418	0.672	2.06E-9
	<34%	0.668	0.489	0.786	5.25E-8	0.66	0.483	0.776	1.14E-9
	34%–66%	0.519	0.38	0.611	6.56E-8	0.513	0.375	0.604	1.87E-9
	≥67%	0.452	0.331	0.532	9.25E-8	0.446	0.327	0.526	4.26E-8
	Valproate	0.830	0.607	0.976	.040	0.820	0.600	0.964	.036
	<34%	0.864	0.632	1.016	.168	0.853	0.624	1.004	.151
	34%–66%	0.837	0.612	0.985	.044	0.827	0.605	0.973	.040
	≥67%	0.730	0.534	0.859	2.33E-5	0.721	0.527	0.849	1.08E-5
Lymphoid leukemia	Without	Reference				Reference			
	Hydralazine	0.420	0.307	0.494	3.37E-5	0.425	0.311	0.500	5.86E-6
	<34%	0	–	–	.985	0.382	0.135	0.491	4.50E-5
	34%–66%	0.849	0.621	0.998	.048	0.859	0.629	1.01	.053
	≥67%	0.740	0.541	0.870	2.65E-6	0.749	0.548	0.881	3.83E-6
	Valproate	0.603	0.441	0.709	4.52E-7	0.610	0.446	0.718	5.58E-7
	<34%	0	–	–	.988	0.284	0.161	0.579	6.86E-5
	34%–66%	1.369	0.991	1.610	.188	1.386	0.978	1.630	.209
	≥67%	0.795	0.582	0.936	.021	0.805	0.589	0.948	.023
Myeloid leukemia	Without	Reference				Reference			
	Hydralazine	0.520	0.38	0.612	7.72E-8	0.526	0.385	0.620	7.98E-8
	<34%	0.866	0.633	1.018	.056	0.877	0.641	1.031	.062
	34%–66%	0	–	–	.993	0.482	0.161	0.597	6.45E-8
	≥67%	0.549	0.402	0.646	6.24E-8	0.556	0.407	0.654	9.03E-8
	Valproate	0.895	0.655	1.053	.176	0.906	0.663	1.066	.196
	<34%	1.165	0.852	1.370	.287	1.179	0.863	1.387	.319
	34%–66%	0.678	0.496	0.917	.001	0.686	0.502	0.928	.001
	≥67%	0.591	0.432	0.695	8.01E-6	0.598	0.437	0.704	5.29E-7
Leukemia of unspecified cell type	Without	Reference				Reference			
	Hydralazine	0.565	0.413	0.665	6.84E-7	0.558	0.408	0.657	7.89E-7
	<34%	0.588	0.43	0.691	8.25E-7	0.581	0.425	0.683	9.22E-7
	34%–66%	0.666	0.487	0.783	5.77E-7	0.658	0.481	0.773	6.03E-7
	≥67%	0.332	0.242	0.390	3.10E-7	0.328	0.239	0.385	3.40E-7
	Valproate	0.878	0.642	1.033	.135	0.867	0.634	1.020	.121
	<34%	0.914	0.669	1.075	.160	0.903	0.661	1.062	.144
	34%–66%	0.920	0.673	1.083	.175	0.909	0.665	1.070	.157
	≥67%	0.713	0.522	0.929	.012	0.704	0.516	0.918	.011
MPN-like neoplasm	Without	Reference				Reference			
	Hydralazine	0.536	0.392	0.631	7.26E-11	0.529	0.387	0.623	5.20E-10
	<34%	0.558	0.408	0.656	8.82E-11	0.551	0.403	0.648	7.26E-10
	34%–66%	0.541	0.396	0.637	7.04E-11	0.534	0.391	0.629	5.58E-10
	≥67%	0.472	0.345	0.555	6.83E-11	0.466	0.341	0.548	1.24E-10
	Valproate	0.841	0.615	0.989	.043	0.831	0.608	0.977	.039
	<34%	0.801	0.586	0.942	.005	0.791	0.579	0.931	.004
	34%–66%	0.946	0.692	1.113	.169	0.934	0.684	1.099	.167
	≥67%	0.761	0.557	0.895	7.80E-8	0.752	0.55	0.884	1.04E-9
Paraproteinemia	Without	Reference				Reference			
	Hydralazine	0.537	0.393	0.632	5.28E-7	0.53	0.388	0.624	6.62E-8
	<34%	0.621	0.454	0.730	3.03E-7	0.613	0.448	0.721	5.04E-8
	34%–66%	0.543	0.397	0.638	5.99E-7	0.536	0.392	0.630	6.88E-8
	≥67%	0.315	0.231	0.371	4.81E-7	0.311	0.228	0.366	4.82E-8
	Valproate	0.771	0.564	0.906	.001	0.762	0.557	0.895	5.54E-6
	<34%	0.802	0.587	0.944	.020	0.792	0.580	0.932	.018
	34%–66%	0.778	0.569	0.915	.007	0.769	0.562	0.904	.006
	≥67%	0.678	0.496	0.798	5.11E-8	0.67	0.490	0.788	4.85E-9
Other polycythemia	Without	Reference				Reference			
	Hydralazine	0.751	0.549	0.883	6.11E-10	0.76	0.556	0.894	7.02E-9
	<34%	0.742	0.542	0.872	6.27E-10	0.751	0.549	0.883	8.11E-9
	34%–66%	0.772	0.564	0.908	5.74E-10	0.782	0.571	0.919	7.34E-9
	≥67%	0.740	0.541	0.870	5.22E-10	0.749	0.548	0.881	6.26E-9
	Valproate	0.849	0.621	1.009	.062	0.859	0.629	1.021	.069
	<34%	0.884	0.647	1.004	.097	0.895	0.655	1.053	.108
	34%–66%	0.871	0.637	1.024	.086	0.882	0.645	1.037	.096
	≥67%	0.723	0.529	0.851	7.80E-9	0.732	0.536	0.862	8.34E-9

a The 1^st^ endpoint in the cohort was defined as the date to stop follow-up when the diagnosis of any HN was made for a subject; while the 2^nd^ endpoint was defined as the end of tracking when follow-up of all the subjects was stopped.

b DDD for HDZ = 300 mg/day, for VAL = 2,100 mg/day. 44,196 subjects who did not receive hydralazine or valproate enrolled in analysis. A total of 11,049 subjects received hydralazine, of which 5,311 received less than 34% DDD, of which 3,646 with 34%–66% DDD, of which 2,092 received more than 67% DDD. A total of 11,049 subjects received valproate, of which 5,307 received less than 34% DDD, of which 3,649 with 34%–66% DDD, of which 2,093 received more than 67% DDD.

Adjusted HR, adjusted hazard ratio; CI, confidence interval.

The Kaplan–Meier curves of the cumulative incidence of HN over the 15-year follow-up period showed a minor difference but did not reach significance between the HDZ and VAL exposure groups in contrast to the reference group (log-rank test *p* = .033). A lower cumulative incidence in the HDZ group (*p* <.001) in comparison with the reference group was revealed, and it was less significant in the VAL group vs. reference group and HDZ group vs. VAL group (log-rank test *p* = .067 and.075, respectively) (**Figure 2B**).

**Figure 3:**
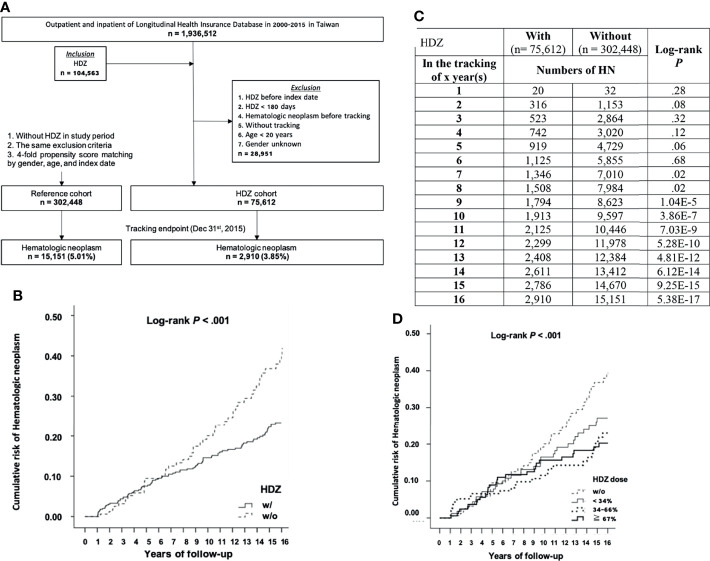
A cohort study focusing on the association of HN incidence reduction with HDZ. **(A)** A flowchart showing sample-subject selection for the cohort study. **(B, C)** The Kaplan–Meier curve for cumulative incidence of HN with log-rank test. **(D)** A Kaplan–Meier curve for cumulative incidence of HN stratified by HDZ dose (% to DDD set as 100 mg per day) with the log-rank test.

We decided to run a further cohort study focusing on HDZ (100 mg per day) at a stratified and lower level of dose range according to WHO DDD guidance. In this study, the subjects (75,612) matching the inclusion criteria in the HDZ group and 302,448 (four-fold) subjects without exposure of HDZ randomly selected for the reference group were compared (**Figure 3A**). Among them, 2,910 subjects (3.85%) were diagnosed with HN after exposure with HDZ while 15,151 subjects (5.01%) were diagnosed with HN in the reference group (**Figure 3A**). The Kaplan–Meier curve of the HDZ group demonstrated a much lower cumulative incidence than the reference group from the 9^th^ year of tracking (**Figures 3B, C** **)**. A Schoenfeld’s global test with *p* value at 0.9014, higher than 0.05, is not against proportional hazards assumption. The Kaplan–Meier curve for cumulative incidence of HN stratified by HDZ dose was disclosed in **Figure 3D**.

### Association of Each HN Subgroup Incidence With Stratified Doses

At the 1^st^ endpoint, the adjusted HRs of all the subgroups in the HDZ group without stratification of the DDD were significantly lower. In the group of overall HN, as well as the subgroups of other malignant neoplasms of lymphoid and histiocytic tissue, multiple myeloma and immunoproliferative neoplasms, leukemia of unspecified cell type, MPN-like neoplasm, paraproteinemia, and other polycythemia, the adjusted HRs of each dose level under defined stratifications were all significantly lower; the adjusted HRs of Hodgkin’s disease, lymphoid leukemia, and myeloid leukemia subgroups were significantly lower at median- and/or highest-dose levels (**Table 1**). The association of incidence of each subgroup of HN in the VAL exposure group showed dose-dependent significance (**Table 1**).

Focusing on the association study of HN incidence in the HDZ exposure group, subjects receiving HDZ with a dose lower than 34 mg per day showed significantly lower HR in overall HNs and in several subgroups such as multiple myeloma and immunoproliferative neoplasms, lymphoid leukemia, myeloid leukemia, leukemia of unspecified cell type, MPN-like neoplasm, MDS, and paraproteinemia (*p* < 0.001 for each subgroup). All the subgroups mentioned above showed a dose-dependent reduction of HR (**Table 2**).

**Table 2:** Adjusted HR of HN according to subgroup stratified by dose level of HDZ.

Hematologic Neoplasm Subgroup	HDZ, dose(DDD *^b^*)	1^st^ Endpoint^a^				2^nd^ Endpoint^a^			
		Adjusted	95% CI		*p*	Adjusted	95% CI		*p*
		HR				HR			
Overall	Without	Reference							
	With	0.730	0.621	0.793	1.73E-18				
	<34%	0.791	0.578	0.927	3.56E-18				
	34%–66%	0.728	0.530	0.858	2.45E-18				
	≥67%	0.581	0.427	0.684	1.21E-18				
Lymphosarcoma and reticulosarcoma	Without	Reference				Reference			
	With	0.911	0.853	0.973	.027	0.922	0.864	0.985	.030
	<34%	1.031	0.965	1.112	.298	1.044	0.977	1.126	.331
	34%–66%	0.879	0.821	0.940	2.59E-04	0.890	0.831	0.952	.017
	≥67%	0.581	0.543	0.620	6.62E-05	0.588	0.55	0.628	6.83E-05
Hodgkin’s disease	Without	Reference				Reference			
	With	0.893	0.837	0.956	.004	0.904	0.847	0.968	.004
	<34%	0.952	0.892	0.972	.027	0.964	0.903	0.984	.030
	34%–66%	0.876	0.824	0.934	1.26E-04	0.887	0.834	0.946	.003
	≥67%	0.752	0.708	0.810	3.31E-05	0.761	0.717	0.820	2.87E-04
Other malignant neoplasms of lymphoid and histiocytic tissue	Without	Reference				Reference			
	With	0.845	0.797	0.906	6.27E-05	0.835	0.787	0.895	3.59E-05
	<34%	0.936	0.878	0.998	.048	0.925	0.867	0.986	.043
	34%–66%	0.761	0.715	0.816	8.00E-07	0.752	0.706	0.806	6.78E-07
	≥67%	0.742	0.698	0.807	4.13E-06	0.733	0.689	0.797	2.18E-06
Multiple myeloma and immunoproliferative neoplasms	Without	Reference				Reference			
	With	0.816	0.763	0.872	6.29E-13	0.806	0.754	0.861	7.89E-13
	<34%	0.825	0.775	0.880	7.89E-12	0.815	0.766	0.869	9.24E-12
	34%–66%	0.820	0.768	0.876	3.12E-13	0.810	0.759	0.865	4.50E-13
	≥67%	0.779	0.724	0.831	4.67E-12	0.770	0.715	0.821	5.98E-12
Lymphoid leukemia	Without	Reference				Reference			
	With	0.698	0.656	0.749	3.58E-17	0.707	0.664	0.758	4.89E-17
	<34%	0.793	0.741	0.848	5.13E-17	0.803	0.750	0.858	7.23E-17
	34%–66%	0.652	0.608	0.698	6.99E-18	0.660	0.616	0.707	7.00E-18
	≥67%	0.494	0.452	0.531	2.79E-20	0.500	0.458	0.538	3.15E-20
Myeloid leukemia	Without	Reference				Reference			
	With	0.759	0.711	0.813	5.56E-17	0.768	0.720	0.823	3.79E-17
	<34%	0.832	0.782	0.898	2.58E-16	0.842	0.792	0.909	5.29E-17
	34%–66%	0.681	0.634	0.735	6.90E-18	0.689	0.642	0.744	7.18E-18
	≥67%	0.666	0.621	0.720	2.01E-19	0.674	0.629	0.729	2.98E-20
Monocytic leukemia	Without	Reference				Reference			
	With	–	–	–	–	0.862	0.625	1.297	.211
	<34%	–	–	–	–	0.975	0.704	1.486	.289
	34%–66%	–	–	–	–	0.813	0.583	1.031	.135
	≥67%	–	–	–	–	0.771	0.492	0.997	.047
Other specified leukemia	Without	Reference				Reference			
	With	1.086	0.303	1.986	.721	1.099	0.307	2.011	.802
	<34%	1.642	0.562	2.111	.521	1.662	0.569	2.137	.580
	34%–66%	0	–	–	.999	1.374	0.289	1.975	.765
	≥67%	0	–	–	.999	1.077	0.161	1.897	.803
Leukemia of unspecified cell type	Without	Reference				Reference			
	With	0.783	0.732	0.839	3.02E-05	0.773	0.723	0.829	2.17E-05
	<34%	0.900	0.848	0.948	2.86E-04	0.889	0.838	0.936	1.01E-04
	34%–66%	0.673	0.634	0.732	3.36E-05	0.665	0.626	0.723	3.15E-05
	≥67%	0.584	0.552	0.637	6.14E-05	0.577	0.545	0.629	5.05E-05
MPN-like neoplasm	Without	Reference				Reference			
	With	0.767	0.718	0.820	6.65E-07	0.758	0.709	0.810	6.26E-07
	<34%	0.817	0.764	0.872	7.80E-07	0.807	0.755	0.861	6.99E-07
	34%–66%	0.781	0.732	0.834	5.46E-07	0.771	0.723	0.824	3.08E-07
	≥67%	0.588	0.551	0.636	2.39E-08	0.581	0.544	0.628	1.09E-08
MDS	Without	Reference				Reference			
	With	0.820	0.764	0.879	6.29E-06	0.810	0.755	0.868	3.19E-06
	<34%	0.871	0.816	0.937	3.86E-05	0.860	0.806	0.926	9.78E-06
	34%–66%	0.860	0.801	0.92	5.48E-05	0.850	0.791	0.909	4.27E-05
	≥67%	0.564	0.522	0.617	3.59E-09	0.557	0.516	0.609	2.85E-09
Paraproteinemia	Without	Reference				Reference			
	With	0.777	0.738	0.849	6.65E-12	0.768	0.729	0.839	5.26E-12
	<34%	0.831	0.775	0.884	5.24E-11	0.821	0.766	0.873	2.73E-11
	34%–66%	0.820	0.762	0.871	6.53E-12	0.810	0.753	0.860	4.01E-12
	≥67%	0.511	0.482	0.555	3.12E-14	0.505	0.476	0.548	1.97E-14
Other polycythemia	Without	Reference				Reference			
	With	0.930	0.871	0.989	.039	0.941	0.882	1.001	.051
	<34%	0.936	0.875	0.996	.047	0.948	0.886	1.008	.059
	34%–66%	0.928	0.866	0.972	.020	0.939	0.877	0.984	.022
	≥67%	0.904	0.852	0.962	.003	0.915	0.863	0.974	.003

a The 1^st^ endpoint in the cohort was defined as the date to stop follow-up when the diagnosis of any HN was made for a subject, while the 2^nd^ endpoint was defined as the end of tracking when follow-up of all the subjects was stopped.

b DDD = 100 mg/day. 302,448 subjects who did not receive hydralazine enrolled in analysis. A total of 75,612 subjects received hydralazine, of which 36,349 received less than 34% DDD, of which 24,952 with 34%–66% DDD, of which 14,311 received more than 67% DDD.

Adjusted HR, adjusted hazard ratio; CI, confidence interval.

The associations of HN incidence for each subgroup at the 2^nd^ endpoint are presented in **Tables 1**, **2**. No significant difference of adjusted HR was found between the 1^st^ and 2^nd^ endpoints, showing that subgroups of HN may not compete with each other. Nevertheless, the probability of coincident occurrence of two subgroups of HN in one subject is very few. For the unassociated subgroups of HN even at high doses, the low disease number can be one of the causes. The subject numbers of most subgroups as mentioned above show sufficient number and significance at the 2^nd^ endpoint.

In addition, subjects who had ever been diagnosed with HTN were selected for analysis since HDZ was often prescribed for HTN. However, no significant association was seen (**Table S5**). The period between start of tracking point and year diagnosed with HN in subjects with HDZ exposure was also significantly longer than those without HDZ (*p* = 2.176E-13, **Table S6**).

### A Sensitivity Analysis for the Association Between the Drugs and HN Incidence

In a cohort study containing VAL, the adjusted HR matching the first endpoint of VAL groups without exclusion, 1^st^-year exclusion, and 3^rd^-year exclusion were 0.877, 0.884, and 0.927, respectively. The adjusted HR in the HDZ groups after exclusion of patients diagnosed with HN in the first 1 and 3 years were 0.720 and 0.756, respectively (both *p* <.001), in comparison to that without exclusion which was 0.714 (*p* < 0.001) (**Table S7**). In the further cohort study focusing on HDZ, the adjusted HRs were 0.741 and 0.720 respectively in the HDZ group after exclusion of HN patients in the first 1 and 5 years, in comparison to that without exclusion was 0.730 (**Table S8**).

In addition, the results obtained from the Fine and Gray competing risk model using all-cause mortality as a competing variable were similar with that obtained from the no competing risk model, indicating that mortality was not a competing variable of HN (**Tables S7**, **S8**).

## Discussion

Over the last decade, several clinical trials have revealed that a combination of HDZ and VAL is a promising therapy for the several hematological malignancies, including mycosis fungoides (22), MDS (23, 24), CTCL (25, 26), AML (27), chronic myeloid leukemia (CML) (28). However, there has been no integral analysis targeting all the HNs as outcomes based on a large health insurance database.

Once HN is diagnosed, the family incurs a heavy financial burden due to a long period of treatment and a low survival rate within 15 years. Our study revealed that prescription of HDZ or even VAL brought beneficial effects to unforeseen patients of particular subgroups of HN. Our results may indicate inadvertent prophylactic benefit. Early prescription may block MPN evolving to AML or BP-MPN. Therefore, studies focusing on screening the population at high incidence of HN are desirable in the future. HDZ may be studied on other high-risk populations of HN, including the following. (1) Patients who underwent solid organ transplantation (29). Several subgroups of HNs tend to occur in solid organ transplant recipients, because most of them need lifelong immunosuppression to prevent organ rejection. Hodgkin lymphoma, non-Hodgkin lymphomas, acute and chronic leukemias, and plasma cell neoplasms (multiple myeloma and plasmacytoma) are more common for those patients. (2) Those exposed to 1,3-butadiene (30). (3) Females with less exposure to female sex hormones (31). Tanaka and colleagues reported that an increased risk of lymphoid neoplasms as well as a shorter menstrual cycle was found in parous women and women with later onset of menarche. (4) Patients with immunodeficiency and autoimmune diseases (32, 33).

Lymphoid leukemia has been classified by stage of maturation (acute lymphoblastic leukemia or chronic lymphocytic leukemia) or according to cell type from which the tumor cells differentiate (T-cell leukemia, B-cell leukemia, NK-cell leukemia) (34). In T-cell leukemia, HDZ treatment was reported to induce apoptosis and cause DNA damage (35). Due to the fact that HDZ acting as a DNMTi (36) decreases the mRNA level of DNMT1 and DNMT3a (37), the role of DNMT1 in B cell maturation (38) and its aberration in B-cell leukemia have been reported (39). A therapeutic significance of HDZ on B-cells as suggested in this retrospective cohort study is conceivable. In addition, with the support by the role of DNMT1 in MDS and myeloid leukemia as reported in a previous review (40) and reports indicating a reduction in mRNA level of DNMTs by HDZ (37) and an abnormally high expression level of DNMTs in cell lines of MDS and myeloid leukemia including AML and CML (41), we suggest that HDZ alone may have enough efficacy to inhibit MDS or myeloid leukemia while previous trials selected a combination of HDZ and VAL (23, 24, 27, 28).

For drugs being considered for repositioning for therapy of a group of diseases such as HNs or other malignancies, a retrospective study like ours could serve as a prior test before a clinical trial protocol is adopted on respective subgroups. We propose that HDZ can be a suitable candidate drug to initiate a phase II clinical trial for treatment of various HNs, instead of starting from a phase I clinical trial. Since epigenetic drugs have a global effect on chromatin, its specific mechanism for a particular disease is not properly defined although several epigenetic drugs have been approved by the FDA. Based on results of our study, chronic prescription of HDZ is significantly associated with reduced HN. However, the adverse effects of repurposing drugs could lead to poor therapeutic compliance and may cause loss-to-follow-up bias in further clinical trials. As a consequence, there should be a reasonable strategy to enhance the therapeutic compliance. Accordingly, we identify that HTN is an independently risk factor for HN (adjusted HR = 1.235, 95% CI = 1.098–1.369, p = 5.267E-18, **Table S9**). As HDZ is an established antihypertensive agent, prescribing a higher-dose HDZ (≥34 mg/day) for HTN participants would ensure better therapeutic compliance than for non-HTN participants. With regard to hypotension-related adverse effects from HDZ prescription, we suggest that prescribing low-dose HDZ (<34 mg/day) is feasible for non-HTN participants, owing to causing mild adverse effects but maintaining preventive efficacy for HNs.

However, many repurposing drugs may not be indicated due to the imbalance of its therapeutic benefits and adverse effects (42). For example, aspirin which is an agent for inflammatory management or preventing heart attack or stroke has been repurposed for multiple myeloma (43), but it may also cause adverse effects such as GI hemorrhage or vascular insufficiency of the intestine (44, 45). Valproate, a psychotic drug for epilepsy, migraine, or seizures, has been repurposed for multiple myeloma (46), but its original indications are excluded from independent risk factors of HNs (**Table S9**). Nelfinavir is an antiviral drug, acting as protease inhibitor and repurposed for HNs by its activity to inhibit the Akt/PKB signaling pathway or to activate ER stress (47). As repurposing nelfinavir for HNs, subjects may suffer from adverse effects caused by the activity of protease inhibitor such as cardiovascular risks (48). In summary, whether a repurposing drug is indicated for HNs depends on the balance of its benefit and harm.

As compared with other drug repurposing for HN therapy, HDZ enables larger potential for repurposing owing to its epigenetics-associated chemopreventive and/or therapeutic efficacy for HNs, as well as its approved indication to manage HTN, an independent risk factor of HNs. In addition to HTN, other independent risk factors of HNs have been identified in **Table S9**, including having gastrojejunal ulcer, Budd-Chiari syndrome, or infection of HBV. If HDZ is prescribed for patients having more risk factors of HNs, it may have better chemopreventive and/or therapeutic efficacy than those having no risk factor of HNs. We suggest that a nationwide retrospective study, as such of this study, could provide evidence for further scientific progress in the medical field.

### Limitations

There are several limitations to this study. Firstly, like many previous NHIRD-based studies, our study was a retrospective cohort study using ICD-9-CM codes, rather than the direct medical records. Therefore, the accuracy of the records may be an issue. Secondly, the pathological stages and severity of HN are unclear in the NHIRD. Thirdly, the NHIRD did not include genetic, educational, habitual, and dietary factors, like smoking and drinking frequency, body mass index, lifestyle, and real income. Fourthly, we could only estimate treatment durations of candidate drug medication by dividing the cumulative doses of individual medications by the DDD. Therefore, a prospective cohort study design would be needed to get more convincing results. Fifthly, the NHIRD is a database sampling of an Asian population; the findings may not be directly applied to other racial populations. Finally, the size of the database may be a limitation as the diagnosed cases of some HN subgroups like monocytic leukemia and other specified leukemia were too few to be analyzed.

## Data Availability Statement

The data analyzed in this study are subject to the following licenses/restrictions: Data are available from the National Health Insurance Research Database (NHIRD) published by the Taiwan National Health Insurance (NHI) Bureau. Due to legal restrictions imposed by the government of Taiwan in relation to the “Personal Information Protection Act,” data cannot be made publicly available. Requests for data can be sent as a formal proposal to the NHIRD (http://nhird.nhri.org.tw). Requests to access these datasets should be directed to https://www.nhi.gov.tw/english/Content_List.aspx?n=A7354F4F704B6377&topn=A7354F4F704B6377 (49).

## Ethics Statement

The studies involving human participants were reviewed and approved by the Institutional Review Board of Tri-Service General Hospital (No. 2-108-05-107, B-109-38). The patients/participants provided their written informed consent to participate in this study.

## Author Contributions

B-HY: English editing, supervising of this work, interpretation of data, overall direction, project administration, and funding acquisition. W-ZL: conducting of the experiments and data acquisition, and interpretation of the data. Y-TC: interpretation of the data. Y-CC: study conceptualization and supervising of this work. C-HC: conducting the experiments and data acquisition. W-CC: interpretation of the data. C-YS: English editing, supervising of this work, interpretation of data, overall direction, project administration, and funding acquisition. All authors contributed to the article and approved the submitted version.

## Funding

The study was funded by the Ministry of National Defense Medical Affairs Bureau (MAB106-036, MAB107-023), the Teh-Tzer Study Group for Human Medical Research Foundation of Taiwan (B1081050), and the Tri-Service General Hospital Foundation (TSGH-B-110012 and TSGH-B-111018).

## Conflict of Interest

Authors W-ZL and C-YS were employed by company Fidelity Regulation Therapeutics Inc.

The remaining authors declare that the research was conducted in the absence of any commercial or financial relationships that could be construed as a potential conflict of interest.

## Publisher’s Note

All claims expressed in this article are solely those of the authors and do not necessarily represent those of their affiliated organizations, or those of the publisher, the editors and the reviewers. Any product that may be evaluated in this article, or claim that may be made by its manufacturer, is not guaranteed or endorsed by the publisher.
